# Potassium *N*-bromo-4-chloro­benzene­sulfonamidate monohydrate

**DOI:** 10.1107/S1600536811023610

**Published:** 2011-06-22

**Authors:** B. Thimme Gowda, Sabine Foro, K. Shakuntala

**Affiliations:** a Department of Chemistry, Mangalore University, Mangalagangotri 574 199, Mangalore, India; b Institute of Materials Science, Darmstadt University of Technology, Petersenstrasse 23, D-64287 Darmstadt, Germany

## Abstract

In the structure of the title compound, K^+^·C_6_H_4_BrClNO_2_S^−^·H_2_O, the K^+^ cation is hepta­coordinated. It is connected to two water O atoms, four sulfonyl O atoms and one Br atom. Further, the sulfonyl and water O atoms in the structure are bridged in a bidentate fashion. The S—N distance of 1.584 (6) Å is consistent with an S—N double bond, The crystal structure is stabilized by inter­molecular O—H⋯N hydrogen bonds.

## Related literature

For our study of the effect of substituents on the structures of *N*-haloaryl­sulfonamides, see: Gowda *et al.* (2007 ▶, 2011**a* ▶,b* ▶); and on the oxidative strengths of *N*-halolaryl­sulfonamides, see: Usha & Gowda (2006 ▶). For similar structures, see: George *et al.* (2000 ▶); Olmstead & Power (1986 ▶). For the preparation of the title compound, see: Gowda & Usha (2003 ▶).
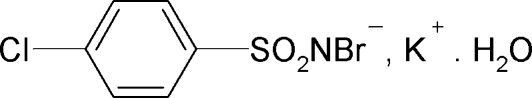


## Experimental

### 

#### Crystal data

K^+^·C_6_H_4_BrClNO_2_S^−^·H_2_O
*M* *_r_* = 326.64
Monoclinic, 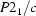

*a* = 15.596 (1) Å
*b* = 10.188 (1) Å
*c* = 6.7649 (7) Å
β = 99.947 (9)°
*V* = 1058.73 (17) Å^3^
*Z* = 4
Mo *K*α radiation
μ = 4.70 mm^−1^
*T* = 293 K
0.34 × 0.34 × 0.30 mm

#### Data collection

Oxford Diffraction Xcalibur diffractometer with a Sapphire CCD detector
Absorption correction: multi-scan (*CrysAlis RED*; Oxford Diffraction, 2009 ▶) *T*_min_ = 0.298, *T*_max_ = 0.333
3796 measured reflections
2147 independent reflections
1984 reflections with *I* > 2σ(*I*)
*R*_int_ = 0.035

#### Refinement

*R*[*F* ^2^ > 2σ(*F* ^2^)] = 0.057
*wR*(*F* ^2^) = 0.156
*S* = 1.19
2147 reflections
134 parameters
2 restraints
H atoms treated by a mixture of independent and constrained refinement
Δρ_max_ = 1.22 e Å^−3^
Δρ_min_ = −0.95 e Å^−3^

### 

Data collection: *CrysAlis CCD* (Oxford Diffraction, 2009 ▶); cell refinement: *CrysAlis RED* (Oxford Diffraction, 2009 ▶); data reduction: *CrysAlis RED*; program(s) used to solve structure: *SHELXS97* (Sheldrick, 2008 ▶); program(s) used to refine structure: *SHELXL97* (Sheldrick, 2008 ▶); molecular graphics: *PLATON* (Spek, 2009 ▶); software used to prepare material for publication: *SHELXL97*.

## Supplementary Material

Crystal structure: contains datablock(s) I, global. DOI: 10.1107/S1600536811023610/ds2118sup1.cif

Structure factors: contains datablock(s) I. DOI: 10.1107/S1600536811023610/ds2118Isup2.hkl

Supplementary material file. DOI: 10.1107/S1600536811023610/ds2118Isup3.cml

Additional supplementary materials:  crystallographic information; 3D view; checkCIF report

## Figures and Tables

**Table 1:** Hydrogen-bond geometry (Å, °)

*D*—H⋯*A*	*D*—H	H⋯*A*	*D*⋯*A*	*D*—H⋯*A*
O3—H31⋯N1^i^	0.82 (2)	2.25 (5)	3.005 (8)	152 (8)
O3—H32⋯N1^ii^	0.82 (2)	2.16 (3)	2.967 (8)	166 (9)

Symmetry codes: (i) 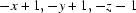; (ii) 
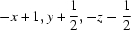
.

**Fig. 1.**
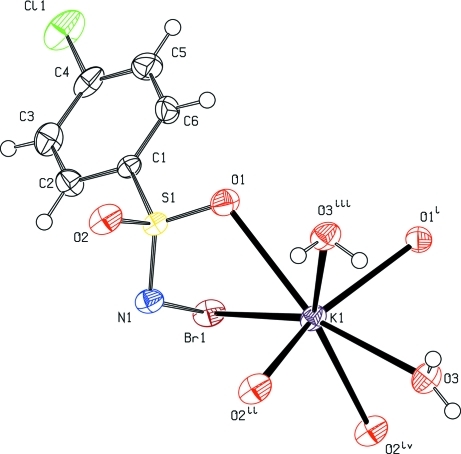
Molecular structure of the title compound, showing the atom labelling scheme for the asymmetric unit and extended to show the coordination geometry for the K+. Displacement ellipsoids are drawn at the 50% probability level. H atoms are represented as small spheres of arbitrary radii.

**Fig. 2.**
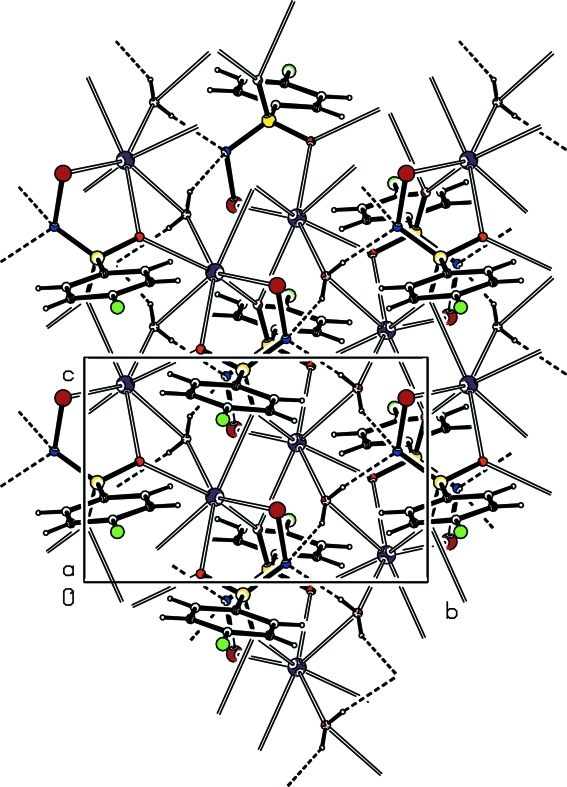
Molecular packing of the title compound with hydrogen bonding shown as dashed lines.

## supplementary crystallographic information

### Comment

To explore the effect of substitution and replacing sodium ion by potassium ion in the solid state structures of *N*-chloroarylsulfonamides (Gowda *et al.*, 2007; 2011**a*,b*; Usha & Gowda, 2006), in the present work, the structure of potassium *N*-bromo-4-chloro- benzenesulfonamidate monohydrate (I) has been determined (Fig. 1). The structure of (I) resembles those of potassium *N*-bromo-2-chloro- benzenesulfonamidate sesquihydrate(II)(Gowda *et al.*, 2011*b*), potassium *N*,4-dichloro-benzenesulfonamidate monohydrate (III) (Gowda *et al.*, 2011*a*), sodium *N*-bromo-4-chloro- benzenesulfonamidate sesquihydrate (IV)(Gowda *et al.*, 2007) and other sodium *N*-chloro-arylsulfonamdes (George *et al.*, 2000; Olmstead & Power, 1986). In particular, there is no interaction between the nitrogen and potassium atom in the molecule. Further, K^+^ is hepta co-ordinated in contrast to hexa co-ordination with Na^+^

K^+^ hepta coordination involves two O atoms from water molecules, four sulfonyl O atoms of *N*-bromo-4-chlorobenzenesulfonamide anions and one Br, in contrast to octahedral coordination of Na^+^ in (IV) by three O atoms of water molecules and by three sulfonyl O atoms of *N*-bromo-4-chloro- benzenesulfonamide anions (Gowda *et al.*, 2007).

The S—N distance of N1—S1, 1.584 (6) Å is consistent with an S—N double bond and is in agreement with the observed values of 1.582 (4) Å in (II), 1.588 (2)Å in (III), and N1—S1, 1.574 (5)Å and N2—S2 1.579 (4)Å in (IV).

K^+^ ion coordination in the structure gives rise to several hydrogen bonding between coordinated water molecules and nitrogen atoms. The packing diagram consists of a two-dimensional polymeric layer running parallel to the *b*-axis (Fig. 2). The molecular packing is stabilized by O3—H31···N1 and O3—H32···N1 hydrogen bonds (Table 1).

### Experimental

The title compound was prepared similar to the literature method (Gowda & Usha, 2003). The purity of the compound was checked by determining its melting point (184 ° C). It was characterized by recording its infrared and NMR spectra.

Yellow prisms of the title compound used in X-ray diffraction studies were obtained from its aqueous solution at room temperature.

### Refinement

The O bound H atoms were located in difference map and later restrained to O—H = 0.82 (2) Å The other H atoms were positioned with idealized geometry using a riding model with C—H = 0.93 Å. All H atoms were refined with isotropic displacement parameters (set to 1.2 times of the *U*_eq_ of the parent atom).

The residual electron-density features are located in the region of Br1. The highest peak and the deepest hole are 1.70 and 0.93 Å from Br1, respectivily.

### Crystal data

**Table:**

K^+^·C_6_H_4_BrClNO_2_S^−^·H_2_O	*F*(000) = 640
*M**_r_* = 326.64	*D*_x_ = 2.049 Mg m^−^^3^
Monoclinic, *P*2_1_/*c*	Mo *K*α radiation, λ = 0.71073 Å
Hall symbol: -P 2ybc	Cell parameters from 2989 reflections
*a* = 15.596 (1) Å	θ = 3.0–27.8°
*b* = 10.188 (1) Å	µ = 4.70 mm^−^^1^
*c* = 6.7649 (7) Å	*T* = 293 K
β = 99.947 (9)°	Prism, yellow
*V* = 1058.73 (17) Å^3^	0.34 × 0.34 × 0.30 mm
*Z* = 4	

### Data collection

**Table:**

Oxford Diffraction Xcalibur diffractometer with a Sapphire CCD detector	2147 independent reflections
Radiation source: fine-focus sealed tube	1984 reflections with *I* > 2σ(*I*)
graphite	*R*_int_ = 0.035
Rotation method data acquisition using ω scans	θ_max_ = 26.4°, θ_min_ = 3.7°
Absorption correction: multi-scan (*CrysAlis RED*; Oxford Diffraction, 2009)	*h* = −19→17
*T*_min_ = 0.298, *T*_max_ = 0.333	*k* = −5→12
3796 measured reflections	*l* = −8→6

### Refinement

**Table:**

Refinement on *F*^2^	Secondary atom site location: difference Fourier map
Least-squares matrix: full	Hydrogen site location: inferred from neighbouring sites
*R*[*F*^2^ > 2σ(*F*^2^)] = 0.057	H atoms treated by a mixture of independent and constrained refinement
*wR*(*F*^2^) = 0.156	*w* = 1/[σ^2^(*F*_o_^2^) + (0.0614*P*)^2^ + 8.645*P*] where *P* = (*F*_o_^2^ + 2*F*_c_^2^)/3
*S* = 1.19	(Δ/σ)_max_ = 0.014
2147 reflections	Δρ_max_ = 1.22 e Å^−^^3^
134 parameters	Δρ_min_ = −0.95 e Å^−^^3^
2 restraints	Extinction correction: *SHELXL97* (Sheldrick, 2008), Fc^*^=kFc[1+0.001xFc^2^λ^3^/sin(2θ)]^-1/4^
Primary atom site location: structure-invariant direct methods	Extinction coefficient: 0.044 (3)

### Special details

**Table:**

Experimental. CrysAlis RED (Oxford Diffraction, 2009) Empirical absorption correction using spherical harmonics, implemented in SCALE3 ABSPACK scaling algorithm.
Geometry. All e.s.d.'s (except the e.s.d. in the dihedral angle between two l.s. planes) are estimated using the full covariance matrix. The cell e.s.d.'s are taken into account individually in the estimation of e.s.d.'s in distances, angles and torsion angles; correlations between e.s.d.'s in cell parameters are only used when they are defined by crystal symmetry. An approximate (isotropic) treatment of cell e.s.d.'s is used for estimating e.s.d.'s involving l.s. planes.
Refinement. Refinement of *F*^2^ against ALL reflections. The weighted *R*-factor *wR* and goodness of fit *S* are based on *F*^2^, conventional *R*-factors *R* are based on *F*, with *F* set to zero for negative *F*^2^. The threshold expression of *F*^2^ > σ(*F*^2^) is used only for calculating *R*-factors(gt) *etc*. and is not relevant to the choice of reflections for refinement. *R*-factors based on *F*^2^ are statistically about twice as large as those based on *F*, and *R*- factors based on ALL data will be even larger.

### Fractional atomic coordinates and isotropic or equivalent isotropic displacement parameters (Å^2^)

**Table:**

	*x*	*y*	*z*	*U*_iso_*/*U*_eq_	
Br1	0.25575 (4)	0.43815 (7)	−0.31985 (10)	0.0299 (3)	
K1	0.44027 (9)	0.61960 (15)	−0.3785 (2)	0.0252 (4)	
Cl1	−0.02963 (13)	0.5964 (3)	0.2758 (3)	0.0534 (6)	
S1	0.34113 (9)	0.53777 (15)	0.0566 (2)	0.0190 (4)	
O1	0.3587 (3)	0.6607 (5)	−0.0369 (7)	0.0310 (11)	
O2	0.4024 (3)	0.5053 (6)	0.2355 (7)	0.0329 (11)	
O3	0.5513 (3)	0.7034 (5)	−0.6370 (8)	0.0318 (11)	
H31	0.572 (5)	0.692 (9)	−0.739 (8)	0.038*	
H32	0.589 (4)	0.753 (7)	−0.578 (12)	0.038*	
N1	0.3390 (4)	0.4126 (6)	−0.0831 (8)	0.0258 (12)	
C1	0.2367 (4)	0.5577 (6)	0.1250 (9)	0.0197 (12)	
C2	0.1965 (4)	0.4475 (7)	0.1854 (11)	0.0278 (14)	
H2	0.2239	0.3662	0.1914	0.033*	
C3	0.1148 (5)	0.4602 (8)	0.2370 (11)	0.0341 (16)	
H3	0.0867	0.3879	0.2806	0.041*	
C4	0.0755 (4)	0.5829 (8)	0.2223 (10)	0.0306 (15)	
C5	0.1156 (5)	0.6906 (8)	0.1639 (11)	0.0371 (17)	
H5	0.0885	0.7721	0.1573	0.044*	
C6	0.1977 (5)	0.6771 (7)	0.1141 (10)	0.0286 (14)	
H6	0.2260	0.7499	0.0733	0.034*	

### Atomic displacement parameters (Å^2^)

**Table:**

	*U*^11^	*U*^22^	*U*^33^	*U*^12^	*U*^13^	*U*^23^
Br1	0.0227 (4)	0.0383 (5)	0.0267 (4)	−0.0011 (3)	−0.0019 (3)	−0.0082 (3)
K1	0.0199 (7)	0.0332 (8)	0.0234 (7)	0.0037 (5)	0.0062 (5)	0.0010 (6)
Cl1	0.0198 (9)	0.0954 (18)	0.0479 (11)	0.0083 (10)	0.0137 (8)	0.0025 (11)
S1	0.0136 (7)	0.0251 (8)	0.0178 (7)	−0.0009 (5)	0.0013 (5)	−0.0026 (5)
O1	0.032 (3)	0.029 (2)	0.034 (3)	−0.009 (2)	0.014 (2)	−0.005 (2)
O2	0.019 (2)	0.052 (3)	0.024 (2)	0.006 (2)	−0.0043 (18)	−0.003 (2)
O3	0.023 (2)	0.042 (3)	0.032 (3)	−0.005 (2)	0.008 (2)	0.000 (2)
N1	0.021 (3)	0.032 (3)	0.024 (3)	0.005 (2)	0.002 (2)	−0.003 (2)
C1	0.018 (3)	0.025 (3)	0.016 (3)	0.000 (2)	0.001 (2)	−0.002 (2)
C2	0.020 (3)	0.027 (3)	0.037 (4)	0.002 (2)	0.007 (3)	0.002 (3)
C3	0.028 (4)	0.040 (4)	0.036 (4)	−0.010 (3)	0.009 (3)	0.002 (3)
C4	0.016 (3)	0.056 (5)	0.020 (3)	0.003 (3)	0.003 (2)	0.000 (3)
C5	0.036 (4)	0.040 (4)	0.036 (4)	0.015 (3)	0.010 (3)	0.002 (3)
C6	0.029 (3)	0.026 (3)	0.033 (3)	0.002 (3)	0.012 (3)	0.006 (3)

### Geometric parameters (Å, °)

**Table:**

Br1—N1	1.897 (5)	O2—K1^ii^	2.784 (5)
Br1—K1	3.5001 (16)	O2—K1^vi^	2.827 (5)
K1—O1^i^	2.704 (5)	O3—K1^i^	2.818 (6)
K1—O2^ii^	2.784 (5)	O3—K1^v^	3.295 (6)
K1—O3	2.799 (5)	O3—H31	0.82 (2)
K1—O3^iii^	2.818 (6)	O3—H32	0.82 (2)
K1—O2^iv^	2.827 (5)	C1—C6	1.356 (9)
K1—O1	2.855 (5)	C1—C2	1.382 (9)
K1—O3^v^	3.295 (6)	C2—C3	1.384 (10)
K1—N1	3.468 (6)	C2—H2	0.9300
Cl1—C4	1.745 (7)	C3—C4	1.388 (11)
S1—O2	1.446 (5)	C3—H3	0.9300
S1—O1	1.450 (5)	C4—C5	1.355 (11)
S1—N1	1.584 (6)	C5—C6	1.386 (10)
S1—C1	1.780 (6)	C5—H5	0.9300
O1—K1^iii^	2.704 (5)	C6—H6	0.9300
			
N1—Br1—K1	73.28 (18)	O2—S1—C1	108.1 (3)
O1^i^—K1—O2^ii^	147.39 (17)	O1—S1—C1	105.3 (3)
O1^i^—K1—O3	78.43 (15)	N1—S1—C1	108.7 (3)
O2^ii^—K1—O3	75.72 (16)	S1—O1—K1^iii^	130.8 (3)
O1^i^—K1—O3^iii^	84.28 (16)	S1—O1—K1	111.7 (3)
O2^ii^—K1—O3^iii^	71.01 (15)	K1^iii^—O1—K1	101.36 (15)
O3—K1—O3^iii^	77.42 (11)	S1—O2—K1^ii^	144.2 (3)
O1^i^—K1—O2^iv^	88.05 (16)	S1—O2—K1^vi^	133.0 (3)
O2^ii^—K1—O2^iv^	99.40 (13)	K1^ii^—O2—K1^vi^	80.60 (12)
O3—K1—O2^iv^	66.52 (15)	K1—O3—K1^i^	99.95 (16)
O3^iii^—K1—O2^iv^	143.94 (16)	K1—O3—K1^v^	72.59 (13)
O1^i^—K1—O1	87.38 (13)	K1^i^—O3—K1^v^	132.42 (18)
O2^ii^—K1—O1	105.97 (15)	K1—O3—H31	149 (6)
O3—K1—O1	150.63 (16)	K1^i^—O3—H31	84 (6)
O3^iii^—K1—O1	75.66 (14)	K1^v^—O3—H31	82 (6)
O2^iv^—K1—O1	139.19 (15)	K1—O3—H32	110 (6)
O1^i^—K1—O3^v^	148.56 (15)	K1^i^—O3—H32	102 (6)
O2^ii^—K1—O3^v^	60.31 (15)	K1^v^—O3—H32	125 (6)
O3—K1—O3^v^	107.41 (13)	H31—O3—H32	98 (9)
O3^iii^—K1—O3^v^	127.12 (10)	S1—N1—Br1	109.5 (3)
O2^iv^—K1—O3^v^	67.58 (15)	S1—N1—K1	83.6 (2)
O1—K1—O3^v^	98.14 (14)	Br1—N1—K1	75.12 (18)
O1^i^—K1—N1	120.12 (14)	C6—C1—C2	121.5 (6)
O2^ii^—K1—N1	89.06 (15)	C6—C1—S1	120.7 (5)
O3—K1—N1	159.76 (16)	C2—C1—S1	117.8 (5)
O3^iii^—K1—N1	110.52 (14)	C1—C2—C3	118.9 (6)
O2^iv^—K1—N1	103.80 (15)	C1—C2—H2	120.6
O1—K1—N1	46.50 (13)	C3—C2—H2	120.6
O3^v^—K1—N1	52.68 (13)	C2—C3—C4	118.7 (7)
O1^i^—K1—Br1	98.17 (12)	C2—C3—H3	120.7
O2^ii^—K1—Br1	114.30 (12)	C4—C3—H3	120.7
O3—K1—Br1	147.31 (12)	C5—C4—C3	122.0 (7)
O3^iii^—K1—Br1	135.01 (11)	C5—C4—Cl1	119.6 (6)
O2^iv^—K1—Br1	80.94 (11)	C3—C4—Cl1	118.4 (6)
O1—K1—Br1	59.70 (10)	C4—C5—C6	118.8 (7)
O3^v^—K1—Br1	59.85 (9)	C4—C5—H5	120.6
N1—K1—Br1	31.60 (9)	C6—C5—H5	120.6
O2—S1—O1	114.6 (3)	C1—C6—C5	120.1 (7)
O2—S1—N1	105.0 (3)	C1—C6—H6	120.0
O1—S1—N1	114.9 (3)	C5—C6—H6	120.0
			
N1—Br1—K1—O1^i^	−137.2 (2)	O3^iii^—K1—O3—K1^v^	125.32 (11)
N1—Br1—K1—O2^ii^	39.8 (2)	O2^iv^—K1—O3—K1^v^	−55.11 (13)
N1—Br1—K1—O3	141.5 (3)	O1—K1—O3—K1^v^	149.3 (3)
N1—Br1—K1—O3^iii^	−47.2 (2)	O3^v^—K1—O3—K1^v^	0.0
N1—Br1—K1—O2^iv^	136.1 (2)	N1—K1—O3—K1^v^	9.7 (4)
N1—Br1—K1—O1	−55.1 (2)	Br1—K1—O3—K1^v^	−60.9 (2)
N1—Br1—K1—O3^v^	66.8 (2)	O2—S1—N1—Br1	177.0 (3)
O2—S1—O1—K1^iii^	−27.2 (5)	O1—S1—N1—Br1	−56.3 (4)
N1—S1—O1—K1^iii^	−148.9 (3)	C1—S1—N1—Br1	61.5 (4)
C1—S1—O1—K1^iii^	91.5 (4)	O2—S1—N1—K1	−111.3 (2)
O2—S1—O1—K1	101.4 (3)	O1—S1—N1—K1	15.5 (3)
N1—S1—O1—K1	−20.3 (4)	C1—S1—N1—K1	133.2 (2)
C1—S1—O1—K1	−139.9 (3)	K1—Br1—N1—S1	77.5 (3)
O1^i^—K1—O1—S1	148.80 (19)	O1^i^—K1—N1—S1	−61.2 (3)
O2^ii^—K1—O1—S1	−61.4 (3)	O2^ii^—K1—N1—S1	103.5 (2)
O3—K1—O1—S1	−150.6 (3)	O3—K1—N1—S1	144.3 (4)
O3^iii^—K1—O1—S1	−126.4 (3)	O3^iii^—K1—N1—S1	34.2 (2)
O2^iv^—K1—O1—S1	64.8 (4)	O2^iv^—K1—N1—S1	−157.0 (2)
O3^v^—K1—O1—S1	−0.1 (3)	O1—K1—N1—S1	−9.74 (19)
N1—K1—O1—S1	11.4 (2)	O3^v^—K1—N1—S1	155.9 (3)
Br1—K1—O1—S1	47.7 (2)	Br1—K1—N1—S1	−112.2 (3)
O1^i^—K1—O1—K1^iii^	−68.3 (3)	O1^i^—K1—N1—Br1	51.0 (2)
O2^ii^—K1—O1—K1^iii^	81.47 (19)	O2^ii^—K1—N1—Br1	−144.32 (18)
O3—K1—O1—K1^iii^	−7.7 (4)	O3—K1—N1—Br1	−103.5 (4)
O3^iii^—K1—O1—K1^iii^	16.46 (16)	O3^iii^—K1—N1—Br1	146.39 (17)
O2^iv^—K1—O1—K1^iii^	−152.3 (2)	O2^iv^—K1—N1—Br1	−44.9 (2)
O3^v^—K1—O1—K1^iii^	142.81 (15)	O1—K1—N1—Br1	102.4 (2)
N1—K1—O1—K1^iii^	154.3 (3)	O3^v^—K1—N1—Br1	−91.9 (2)
Br1—K1—O1—K1^iii^	−169.3 (2)	O2—S1—C1—C6	112.9 (6)
O1—S1—O2—K1^ii^	−88.7 (6)	O1—S1—C1—C6	−10.0 (6)
N1—S1—O2—K1^ii^	38.3 (6)	N1—S1—C1—C6	−133.7 (6)
C1—S1—O2—K1^ii^	154.2 (5)	O2—S1—C1—C2	−68.3 (6)
O1—S1—O2—K1^vi^	66.9 (5)	O1—S1—C1—C2	168.8 (5)
N1—S1—O2—K1^vi^	−166.1 (4)	N1—S1—C1—C2	45.1 (6)
C1—S1—O2—K1^vi^	−50.2 (5)	C6—C1—C2—C3	−0.4 (10)
O1^i^—K1—O3—K1^i^	−16.53 (16)	S1—C1—C2—C3	−179.2 (5)
O2^ii^—K1—O3—K1^i^	−176.5 (2)	C1—C2—C3—C4	1.2 (11)
O3^iii^—K1—O3—K1^i^	−103.2 (2)	C2—C3—C4—C5	−1.6 (11)
O2^iv^—K1—O3—K1^i^	76.40 (17)	C2—C3—C4—Cl1	176.9 (6)
O1—K1—O3—K1^i^	−79.2 (3)	C3—C4—C5—C6	1.1 (11)
O3^v^—K1—O3—K1^i^	131.51 (17)	Cl1—C4—C5—C6	−177.4 (6)
N1—K1—O3—K1^i^	141.2 (4)	C2—C1—C6—C5	−0.1 (10)
Br1—K1—O3—K1^i^	70.6 (3)	S1—C1—C6—C5	178.6 (5)
O1^i^—K1—O3—K1^v^	−148.04 (15)	C4—C5—C6—C1	−0.2 (11)
O2^ii^—K1—O3—K1^v^	52.01 (12)		

Symmetry codes: (i) *x*, −*y*+3/2, *z*−1/2; (ii) −*x*+1, −*y*+1, −*z*; (iii) *x*, −*y*+3/2, *z*+1/2; (iv) *x*, *y*, *z*−1; (v) −*x*+1, −*y*+1, −*z*−1; (vi) *x*, *y*, *z*+1.

### Hydrogen-bond geometry (Å, °)

**Table:**

*D*—H···*A*	*D*—H	H···*A*	*D*···*A*	*D*—H···*A*
O3—H31···N1^v^	0.82 (2)	2.25 (5)	3.005 (8)	152 (8)
O3—H32···N1^vii^	0.82 (2)	2.16 (3)	2.967 (8)	166 (9)

Symmetry codes: (v) −*x*+1, −*y*+1, −*z*−1; (vii) −*x*+1, *y*+1/2, −*z*−1/2.
